# Human amniotic epithelial cells improve fertility in an intrauterine adhesion mouse model

**DOI:** 10.1186/s13287-019-1368-9

**Published:** 2019-08-14

**Authors:** Boning Li, Qiuwan Zhang, Junyan Sun, Dongmei Lai

**Affiliations:** 1 International Peace Maternity and Child Health Hospital, School of Medicine, Shanghai Jiao Tong University, Shanghai, 200030 China; 2 Shanghai Key Laboratory of Embryo Original Diseases, Shanghai, 200030 China; 3 Shanghai Municipal Key Clinical Speciality, Shanghai, 20030 China

**Keywords:** Human amniotic epithelial cells, Intrauterine adhesion, Asherman syndrome, Autophagy, Fibrosis

## Abstract

**Background:**

Intrauterine adhesion (IUA) is an adhesion of the uterine cavity or cervical canal resulting from damage to the basal layer of the endometrium; this condition is usually accompanied by fibrosis of the endometrium. Previous studies have demonstrated that human amniotic epithelial cells (hAECs) have stem cell characteristics; however, it is unclear whether hAECs have the therapeutic potential to restore fertility after IUA.

**Methods:**

A murine IUA model was established by mechanical injury to the uterus. Then, 10^6^ hAECs were transplanted by intraperitoneal injection. The endometrium thickness, number of glands, and fibrosis area were measured by hematoxylin and eosin (H&E) staining and Masson staining. Molecules (including vWF, VEGF, PCNA, ER, PR, LC3, and p62) related to endometrial angiogenesis, cell proliferation, and autophagy were assayed by IHC staining. Pregnancy outcomes were also evaluated. Finally, hAECs were cocultured with human endometrial mesenchymal stem cells (hEnSCs) damaged by H_2_O_2_ to verify the paracrine effect on endometrial stromal cells in vitro.

**Results:**

The IUA uterine cavity presented with adhesion and even atresia, accompanied by a thinner endometrium, fewer glands, increased fibrosis area, and fewer microvessels. However, hAECs significantly improved the uterine structure after IUA. After hAEC treatment, the endometrium was thicker, the number of endometrial glands was increased, fibrosis was reduced, and more microvessels were generated. The expression levels of VEGF, PCNA, and ER were increased in the hAEC-treated endometrium, indicating improvements in angiogenesis and stromal cell proliferation. hAECs also increased pregnancy outcomes in IUA mice, and the pregnancy rate and fetus number increased. Furthermore, we observed altered autophagy in the IUA uterine model, and hAEC transplantation upregulated autophagy. An in vitro study showed that hAECs activated autophagy in (hEnSCs) treated with H_2_O_2_ in a paracrine manner.

**Conclusions:**

Our results demonstrated that hAECs have the potential to repair the uterus after injury, providing a new strategy for the prevention and treatment of Asherman syndrome.

## Introduction

Intrauterine adhesion (IUA) is a consequence of endometrial trauma that leads to the complete or partial obstruction of the uterine cavity or cervical canal. IUA can also be defined as Asherman syndrome, which is characterized by symptoms such as hypomenorrhea, menopause, pelvic pain, recurrent abortion, or infertility [1]. In general, a common cause of IUA is artificial trauma to the uterine cavity during operation procedures, such as curettage, cesarean section, and hysteromyomectomy, which may injure the endometrial basal layer. Tuberculosis and other infections may result in chronic inflammation of the endometrium, which is also a trigger for adhesion. Additionally, congenital Müllerian anomalies, such as uterus septus, increase the incidence of IUA. Trauma, infection, and genetic susceptibility result in the loss of spontaneous endometrium recovery and angiogenesis, initiating adhesion of the endometrium [2]. The incidence of Asherman syndrome is approximately 1.5%. However, repeat curettage resulting from a miscarriage can increase the incidence of IUA to 39% [3]. Currently, hysteroscopy adhesiolysis is the treatment of choice for Asherman syndrome [4]. However, even after adhesiolysis, patients are still susceptible to pregnancy complications, such as preterm delivery and anomalous placenta development, because of impaired metabolism and angiogenesis in the endometrium [5]. Because two thirds of women with Asherman syndrome have undergone post-abortion/miscarriage curettage, it is necessary to take actions to prevent adhesion after invasive operations in the uterine cavity [6]. This can be accomplished via several methods, such as placing an intrauterine device (IUD)/Foley’s catheter balloon/hyaluronic acid in the uterine cavity or using conjugated estrogen treatment to facilitate endometrium recovery [7–9]. However, a foreign object may give rise to infection risks, and various therapies lack evidence from large-scale randomized clinical trials and lack further follow-up for pregnancy outcomes [10, 11]. Hence, to lower the incidence of Asherman syndrome, efficient efforts to prevent adhesion immediately after exposure to the risk factors would be of great benefit.

Stem cell therapy has been recognized as a potential treatment strategy for IUA. Kilic et al. established a rodent IUA model with trichloroacetic acid and then combined intraperitoneal injection of mesenchymal stem cells (MSCs) with oral estrogen for treatment. These authors showed that MSCs decreased the fibrotic area in the uterus and activated cell proliferation and angiogenesis [12]. Zhang et al. found that in situ injections of induced pluripotent stem cells (iPSCs) could rescue IUA induced by either mechanical injury or lipopolysaccharide (LPS) in mice, thus reducing inflammation and improving fertility [13]. Gan et al. reported that intramuscular injections of human amniotic mesenchymal stromal cells (hAMSCs) could induce endometrium regeneration in rodent IUA models [14]. The efficacy of menstrual blood-derived stromal cells (menSCs) was initially demonstrated in clinical research. Seven severe Asherman syndrome patients were transplanted with their own menSCs. Five of these patients reached the endometrium thickness standard to receive frozen embryo transfer (FET) after transplantation. In the 3-year follow-up, three patients became pregnant spontaneously or through FET [15]. Bone marrow-derived stem cells (BMDSCs) were also proven to be effective in both animal models and clinical trials [16–18].

In recent years, human amniotic epithelial cells (hAECs) derived from the placenta have been shown to have the multiplex differential potential of embryonic stem cells and the immune-regulating potential of adult stem cells. As perinatal stem cells, hAECs have attracted much attention in tissue regeneration because of their low mutation frequency, low immunogenicity, low tumorigenicity, and rich resources [19, 20]. However, whether hAECs could facilitate IUA recovery is unclear.

In this study, we aimed to demonstrate whether hAEC transplantation during endometrial repair in an IUA mouse model could improve reproductive performance.

## Materials and methods

### Culture and preparation of hAECs

hAECs were isolated as described previously [21]. The protocol was approved by the Institutional Ethics Committee of the International Peace Maternity and Child Health Hospital. Human amniotic membranes were obtained from healthy women who provided informed consent. Briefly, the amnion was mechanically dissected into segments, digested with 0.25% trypsin/EDTA (Thermo Fisher Scientific, Waltham, MA, USA) at 37 °C for 25 min, filtered through a 40-μm filter, and then centrifuged for 5 min at 300*g*. Cells were seeded onto 100-mm plates containing Dulbecco’s modified Eagle’s medium (DMEM)/F12 (Gibco, Grand Island, NY, USA) with 10% fetal bovine serum (FBS, Gibco) with 5% CO_2_ at 37 °C. For hAEC transplantation, the hAECs were digested with 0.25% trypsin-EDTA (Biological Industries, Kibbutz BeitHaemek, Israel) at 37 °C and then resuspended in phosphate-buffered solution (PBS) at 10^7^/mL. hAECs used in the experiments had undergone fewer than two passages.

### Animal model

All animal procedures were approved by the Institutional Animal Care and Use Committee of Shanghai and were performed in accordance with the National Research Council Guide for the Care and Use of Laboratory Animals. Six-week-old Balb/c mice were obtained from the Shanghai Experimental Animal Center of the Chinese Academy of Sciences and allowed to adapt to the new environment for at least a week. The mice were maintained in SPF conditions. Vaginal smears were obtained daily at 08:00 am to assess estrus cycles. Mice with consecutive 4-day estrus cycles were used to establish the animal model.

To establish an IUA model, mechanical injury of the uterus was performed at the diestrus stage [14, 17]. The mice were anesthetized by intraperitoneal injection of pentobarbital sodium. The uterus was exposed by an excision in the low midline abdomen. A 7-gauge needle was inserted into the connection between the left and right uterus, and both areas were scratched back and forth carefully until the uterus became hyperemic to the naked eye. Subsequently, the abdominal cavity was closed. The surgical procedure was performed under sterile conditions. The control group did not undergo surgery. For the hAEC-treated group, 100 μL of a 10^7^/mL hAEC (10^6^ hAECs) suspension was injected intraperitoneally according to our previous research [21, 22]. The IUA group received 100 μL of PBS. The same amount of hAEC suspension was injected intraperitoneally on three subsequent days after the operation.

### Histological analysis

Uterus specimens were collected 8 days after the operation. Specimens were fixed in 4% paraformaldehyde, dehydrated, cleared in xylene, and finally embedded in paraffin. The embedded tissues were sliced into 5-μm-thick sections. Hematoxylin and eosin (H&E) staining was used to evaluate the morphological structure of the uterus. Masson staining was applied according to the manufacturer’s instructions (60532ES74, Yeasen, Shanghai, China). The fibrosis area, which was stained pale blue, was evaluated with ImageJ software (NIH, MD, USA).

### Flow cytometry

For hAEC characterization, cells were harvested and incubated with labeled primary antibodies (SSEA4-FITC: 330410, isotype control 401317; CD324-APC: 324108, isotype control 400122; CD146-PE: 361006, isotype control 400114; HLADR-FITC: 11-9956-42, isotype control 11-4724-82; Biolegend, USA) at 4 °C for 30 min. Then, the cells were washed with PBS and analyzed with a BD Accuri C6 (BD Biosciences, NJ, USA).

### Immunofluorescence staining

For hAEC characterization, cells were plated on glass coverslips. After the cell density reached approximately 90%, the cells were fixed with 4% paraformaldehyde for 10 min at room temperature and washed with PBS. The cells were permeabilized with 0.5% Triton X-100 for 20 min, washed with PBS, and blocked with 3% goat serum for 30 min. Then, the cells were incubated with primary antibody (CK18: Boster Biological Technology, China; TRA-1-60: Cell Signaling Technology, USA) overnight at 4 °C. The cells were probed with Alexa 594, and the slides were mounted with medium with DAPI (H1200, Vector Laboratories).

For the cell tracking assay, hAECs were previously labeled with carboxyfluorescein succinimidyl ester (CFSE) according to the manufacturer’s instructions (CFDA SE Cell Proliferation and Cell Tracking Kit, Yeasen), and the IUA model was established in mice. For the CFSE group, the mouse uteruses were mechanically injured; then, 100 μL of 10^7^ CFSE-labeled hAECs/mL was injected into the uterus cavity, and the same amount of hAECs in suspension was injected intraperitoneally for three following days. For the control group, murine uteruses were also injured, but the hAEC suspension was replaced with PBS. After 3 days, the uteruses were collected and fixed with optimal cutting temperature (OCT) compound (Sakura Finetek, Seattle, USA), and 10-μm fresh sections were generated. The slides were mounted with a medium with DAPI. Fluorescence images were obtained with a Leica DMI3000 microscope (Heidelberg, Germany).

### Immunohistochemical analysis

Primary anti-VEGFA (ab52917, Abcam, USA), anti-vWF (PB0273, Boster, Wuhan, China), anti-PCNA (BM0104, Boster, Wuhan, China), anti-LC3 (WL01506, Wanlei, Shenyang, China), anti-p62 (WL02385, Wanlei, Shenyang, China), anti-ERα (ab32063, Abcam, USA), and anti-PR (sc-810, Santa Cruz, TX, USA) antibodies were diluted in 0.5% goat serum in PBS. The sections were heated in a microwave in sodium citrate solution for antigen recovery and pretreated with 0.3% H_2_O_2_ in methanol to quench endogenous peroxidase activity. Then, the samples were incubated with 3% goat serum to block nonspecific antibody binding sites. Subsequently, the samples were incubated with primary antibodies at 4 °C overnight. Immunoreactivity was visualized using a Mouse and Rabbit Specific HRP/DAB Detection IHC kit (ab64264, Abcam) according to the manufacturer’s instructions.

### Fertility assay

Nine days post-procedure, female mice were mated with male Balb/c mice whose fertility had been verified  (8 weeks old). The morning of vaginal plug presence was considered to be gestational day 0.5. All female mice presented vaginal plugs in two estrus cycles. At gestation day 9, the female mice were sacrificed for uterus examination.

### Coculture assay

Human endometrial mesenchymal stem cells (hEnSCs) were isolated from the menstrual blood of a 40-year-old Chinese woman according to a protocol previously described [23]. Briefly, menstrual blood was collected, and hEnSCs were acquired by density-gradient centrifugation. The procedure was approved by the Institutional Ethics Committee of the International Peace Maternity and Child Health Hospital (Shanghai, China). hEnSCs were cultured in Chang Medium (S-Evans Biosciences, Hangzhou, China) with 5% CO_2_ at 37 °C. For western blot assay and morphological assessment, 2 × 10^5^ hEnSCs were seeded in the lower compartment of a 6-well plate. H_2_O_2_ was added to a terminal concentration of 1 μM to simulate IUA characteristics in vitro according to previous reports [24]. After 2.5 h, the former medium was discarded and replaced with fresh medium. For the hAEC-treated group, 10^5^ hAECs were seeded onto a Transwell insert with a 0.4-μm pore size membrane (Corning) and placed above the lower compartment for coculture. After 24 h, the hEnSCs were collected for protein extraction.

### Cell viability assessment

For the cell viability assay, hEnSCs were seeded onto 24-well plates at 5 × 10^4^ cells per well. Cells in the injury group were treated with 1 μM H_2_O_2_ for 2.5 h. Then, the former medium was discarded and replaced with fresh medium. After 24 h, cell viability was assessed using a Cell Counting Kit-8 (CCK8) (Yeasen) according to the manufacturer’s instructions.

### Western blot analysis

Proteins were extracted from hEnSCs samples using RIPA buffer (Beyotime, Shanghai, China), and the protein concentrations were quantified using a Pierce BCA Protein Assay Kit (23225, Thermo Fisher Scientific, USA). Proteins were separated in polyacrylamide gel and transferred to the PVDF membranes. Anti-p62 (88588, Cell Signaling Technology, USA), anti-LC3 (WL01506, Wanlei, Shenyang, China), and anti-β-tubulin (30303ES10, Yeasen, Shanghai, China) were used as primary antibodies. The blots were visualized using an Enhanced Chemiluminescence Kit (New Cell & Molecular Biotech Co., Suzhou, China), and the band intensity was quantified with ImageJ software.

### Statistical analysis

The data are expressed as averages with standard deviations. Student’s *t* test or an ordinary one-way analysis of variance (ANOVA) was used to analyze the data. Significant differences were calculated using GraphPad Prism version 6 (GraphPad Software, La Jolla, USA). Differences were considered statistically significant when *p* < 0.05.

## Results

### hAECs restore endometrial morphology in a mouse model of intrauterine adhesions

**Fig. 1:**
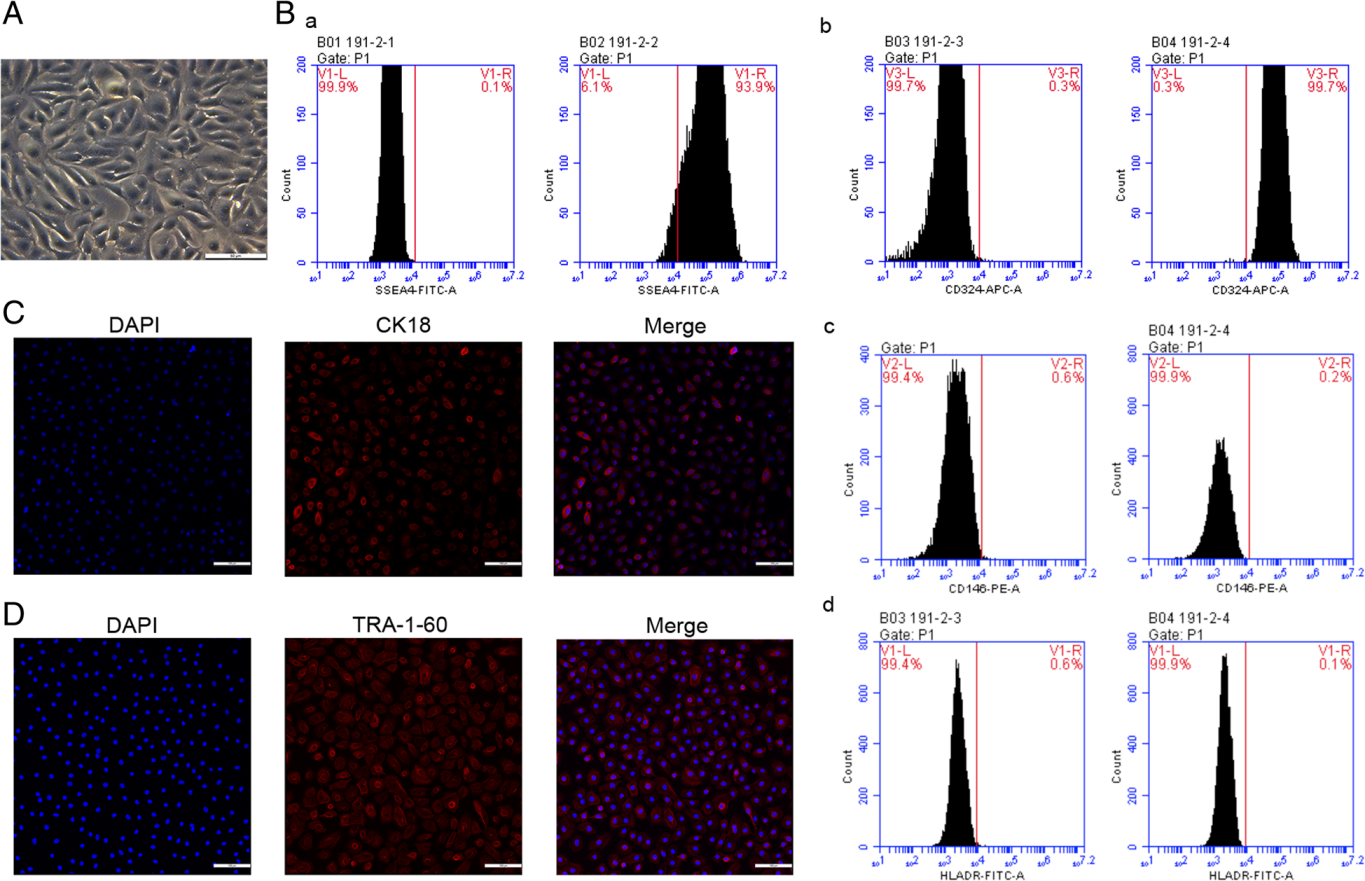
hAECs presented with stem cell characteristics and low immunogenicity. **A** hAECs presented an epithelial morphology under bright-field microscopy. Scale bar = 50 μm. **B** By flow cytometry, hAECs were positive for (a) stem cell marker SSEA4 and (b) epithelial marker CD324 and were negative for (c) mesenchymal markers CD146 and (d) HLA-DR. **C** Immunofluorescence staining for CK18 (an epithelial marker) expression in hAECs. Scale bar = 100 μm. **D** Immunofluorescence staining for TRA-1-60 (a stem cell marker) expression in hAECs. Scale bar = 100 μm

Isolated hAECs presented the cobblestone-like morphology of epithelial cells in vitro (Fig. 1A). Flow cytometry was used to evaluate the characteristics of hAECs. The results showed that hAECs expressed high levels of the stem cell marker SSEA4 and the epithelial marker CD324, while the expression of the mesenchymal marker CD146 and the immunogenicity indicator human leukocyte antigen HLA-DR was lacking (Fig. 1B). Immunofluorescence assays further showed that the epithelial marker CK18 and the stem cell marker TRA-1-60 were both present in hAECs (Fig. 1C, D).

**Fig. 2:**
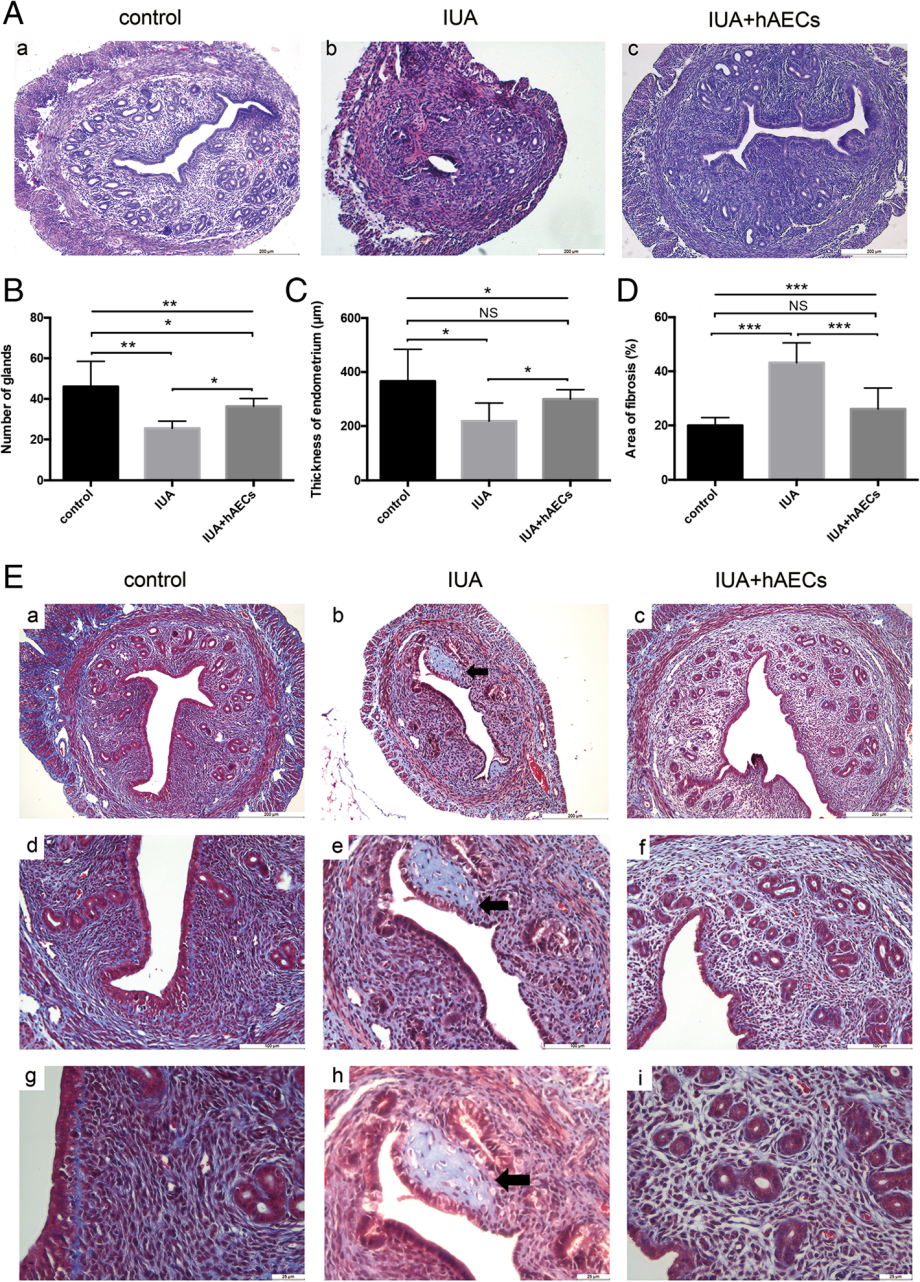
Endometrial morphology in hAECs was restored in a mouse model of intrauterine adhesions. **A** (a) H&E staining showed that the endometrium of a normal mouse was in a contact arrangement, with abundantly scattered glands and columnar epithelium cells lined clearly on the cavity surface. (b) In the IUA model, the uterine cavity was adherent. (c) In the hAEC-treated group, uterine adhesion decreased. Scale bar = 200 μm. **B** Compared with that in the control group, the number of glands decreased in the IUA group, but the hAEC-treated group showed a higher number of glands than the IUA group. **C** The thickness of the endometrium in the IUA group was decreased compared to that in the control group and hAEC-treated group. **D** A larger percent of the fibrosis area of the endometrium was found in the IUA model. The fibrosis area decreased in the hAEC-treated group. **E** Masson staining showed fibrotic tissue (stained pale blue) in the endometrium. A fibrotic mass was found in the damaged endometrium (black arrow pointing) (b, e, h), and the fibrosis area was larger in the IUA group than in the control group (a, d, g) and hAEC-treated group (c, f, i). a–c, scale bar = 200 μm; d–f, scale bar = 100 μm; g–i, scale bar = 25 μm (*n* = 6; *p < 0.05; **p < 0.01; ***p < 0.001; NS, *p* ≥ 0.05)

We created a murine model for IUA using mechanical injury. Mice in the hAEC group were treated with 10^6^ hAECs immediately after the operation and on three subsequent days by intraperitoneal injection. The same volume of PBS was given to the IUA group, and the control group did not receive the operation. Uterus samples were collected 8 days after the operation. According to H&E staining results, compared to the normal murine uterus, the IUA uterine cavity presented adhesion and even atresia. The intact and smooth cavity was damaged (Fig. 2A, (a, b)), the endometrium in IUA mice became thinner (218.46 ± 66.91 μm), and the number of glands decreased (25.50 ± 3.507) (Fig. 2B, C). However, in the hAEC-treated group, the uterine cavity morphology improved (Fig. 2A, (c)), the endometrium thickness increased (299.67 ± 35.29 μm), and more glands appeared (36.33 ± 3.83), almost reaching the values in the control group (366.32 ± 118.07 μm, 46.00 ± 12.51, respectively) (Fig. 2B, C).

Fibrosis is a feature of endometrial adhesion. Masson staining was used to evaluate the extent of fibrosis. Compared with that in the control group (19.98 ± 2.92%), the fibrotic area in the endometrium of IUA model mice was significantly increased (43.18 ± 7.35%), but hAEC treatment led to a remarkable reduction in fibrosis (26.08 ± 7.73%) (Fig. 2D, E).

**Fig. 3:**
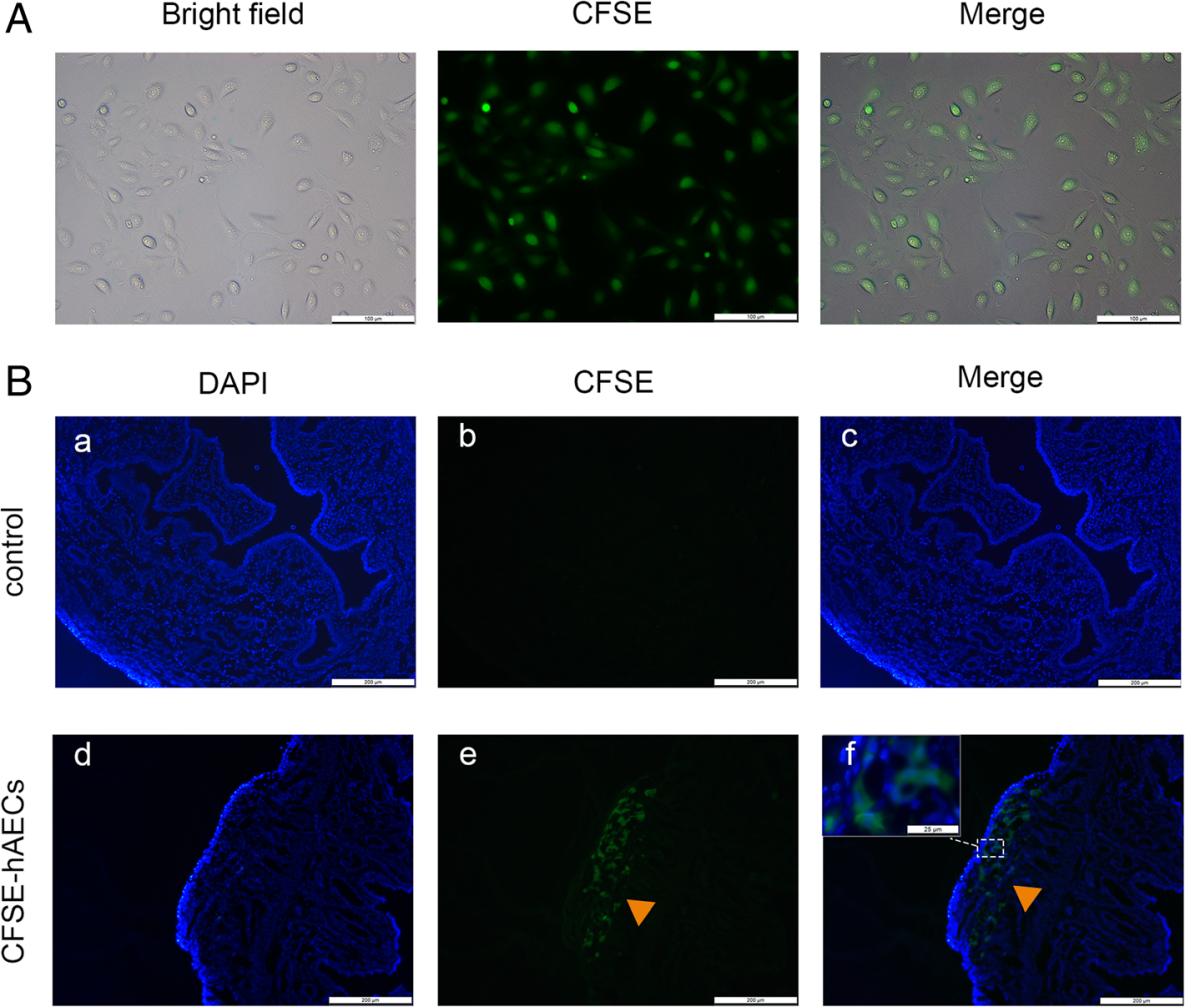
hAECs migrated to the injured uterine in vivo*.* **A** hAECs were labeled with CFSE before transplantation into the mice. The expression rate of green fluorescence staining was 100%. Scale bar = 100 μm. **B** According to immunostaining, CFSE-labeled hAECs (arrowheads) engrafted in the injured uterine at 3 days after hAEC transplantation, while no fluorescence-stained cells were detected in IUA mice without hAEC transplantation. a–f, scale bar = 200 μm; inset, scale bar = 25 μm

To determine whether hAECs could migrate to the injured uterine, hAECs were labeled with green fluorescent CFSE and transplanted into the IUA model (Fig. 3A). Immunostaining demonstrated that green fluorescent cells were detected in hAEC-treated IUA uteruses at 3 days after transplantation, while no green fluorescence was found in the control IUA uteruses (negative control, treated with PBS) (Fig. 3B).

### hAECs facilitated endometrial angiogenesis and stromal cell proliferation

**Fig. 4:**
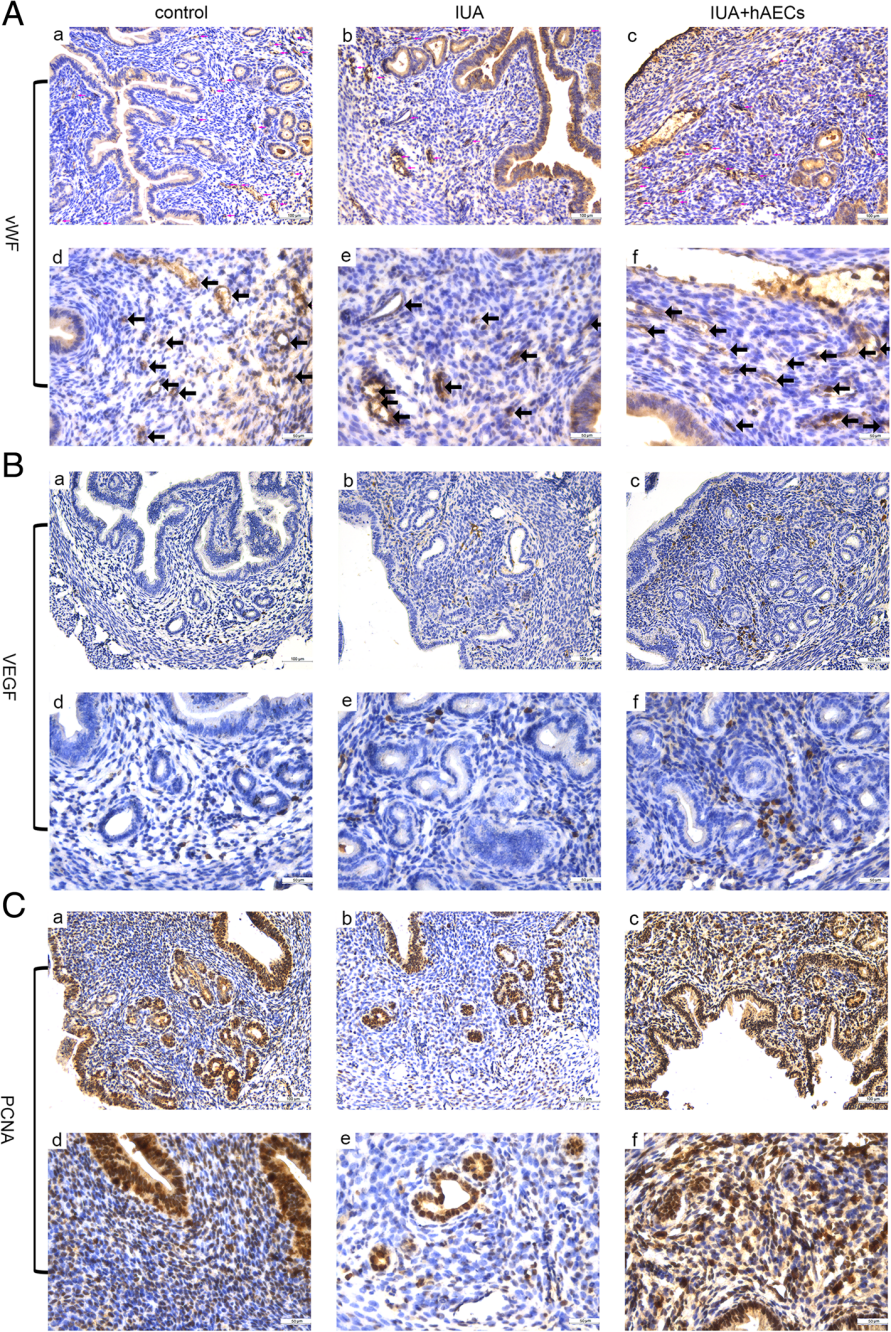
hAECs facilitated endometrial recovery in the IUA mouse model. **A** IHC staining of vWF reflected the MVD of the endometrium. The microvessels, which were vWF-positive, are indicated by arrows in the figure. MVD was reduced in the IUA group and increased in the hAEC-treated group. **B** IHC staining showed that the expression of VEGF was higher in the hAEC-treated group than in the IUA group. **C** The expression of PCNA decreased in the IUA group and reached almost normal levels in the hAEC-treated group. a–c, scale bar = 100 μm; d–f, scale bar = 50 μm

**Fig. 5:**
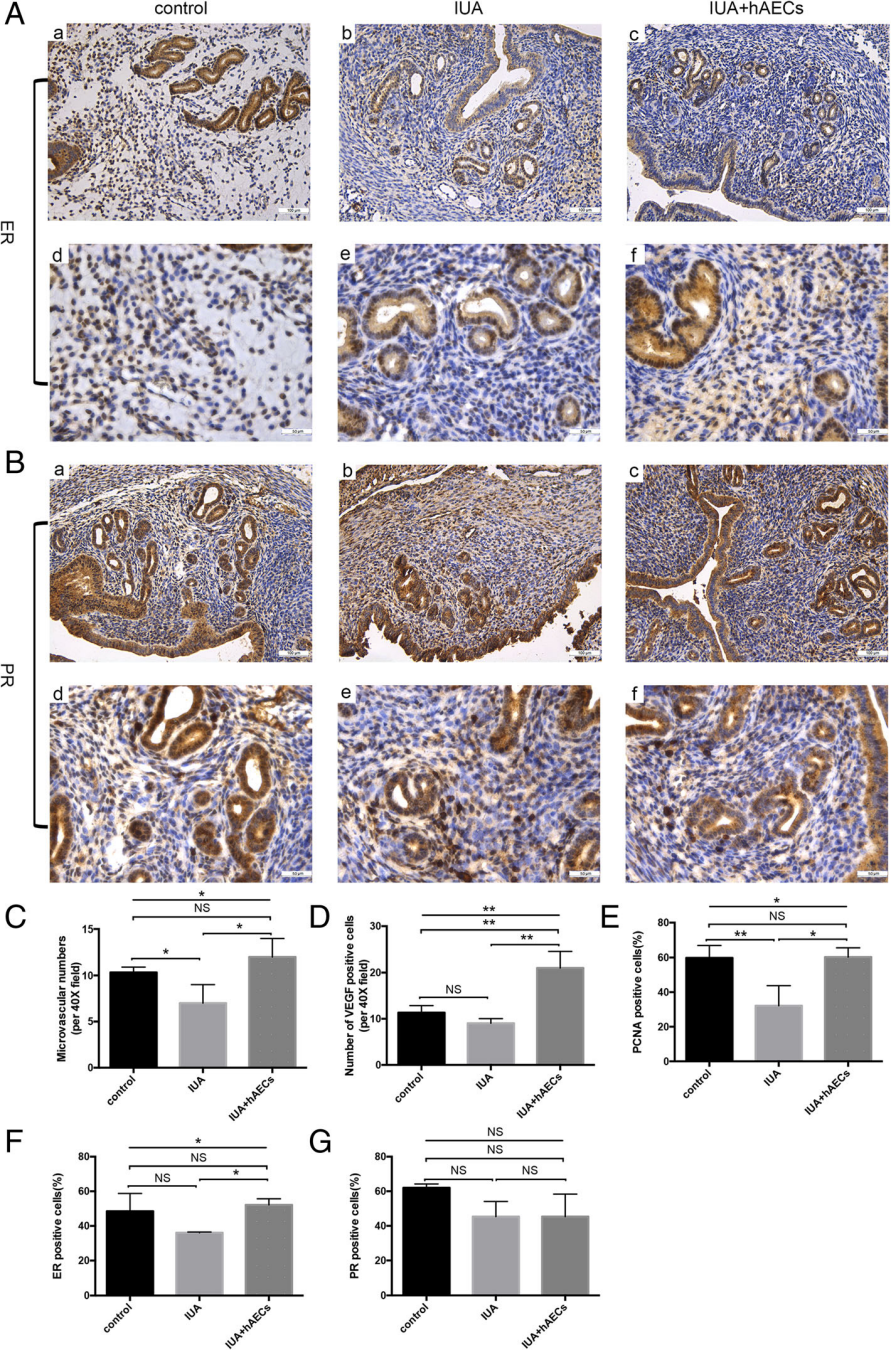
hAECs facilitated endometrial recovery in the IUA mouse model. **A** According to IHC staining, the number of ER-positive cells was higher in the hAEC-treated group than in the IUA group. **B** There was no difference in PR expression among these three groups. **C** VEGF expression was semi-quantified, and the number of positive cells per field was calculated. **D** MVD was valued by counting microvascular vessels, which were vWF-positive. **E**–**G**. PCNA, ER, and PR expression levels were semi-quantified by calculating the percentage of positive cells per field (*p < 0.05; **p < 0.01; ***p < 0.001; NS, *p* ≥ 0.05). a–c, scale bar = 100 μm; d–f, scale bar = 50 μm

IHC staining was used to investigate cell proliferation and angiogenesis in endometrium recovery. To measure the re-establishment of blood supply in the endometrium, we calculated the microvessel density (MVD) using von Willebrand factor (vWF), a highly specific vascular endothelial marker. The results showed that MVD was lower in the IUA group than in the normal control group; however, MVD was increased in the hAEC-treated group (Fig. 4A, Fig. 5C). Additionally, the expression of vascular endothelial growth factor (VEGF), a specific vascular endothelial cell growth-promoting factor, increased significantly in the endometrium of the hAEC-treated group compared with that in the IUA group (Fig. 4B, Fig. 5D).

Proliferating cell nuclear antigen (PCNA) is an objective index to evaluate the cell proliferation state. Quantitative analysis confirmed that PCNA expression decreased in the injured endometrium, while PCNA expression significantly increased in the hAEC-treated group (Fig. 4C, Fig. 5E).

Estrogen receptor (ER) and progesterone receptor (PR) are indicators of endometrial recovery in IUA [25]. Our results showed that ER expression was significantly increased in the hAEC-treated group compared with that in the IUA group, while the expression of PR changed little among the three groups (Fig. 5A, B, F, G).

### hAECs ameliorated pregnancy outcomes in the IUA mouse model

The IUA mice were mated with confirmed fertile male mice to assess the function of the injured uterus. The female mice were euthanized at 8.5 days after the presence of the vaginal plug, and the uterine horns were examined for the numbers and sizes of the fetuses.

**Fig. 6:**
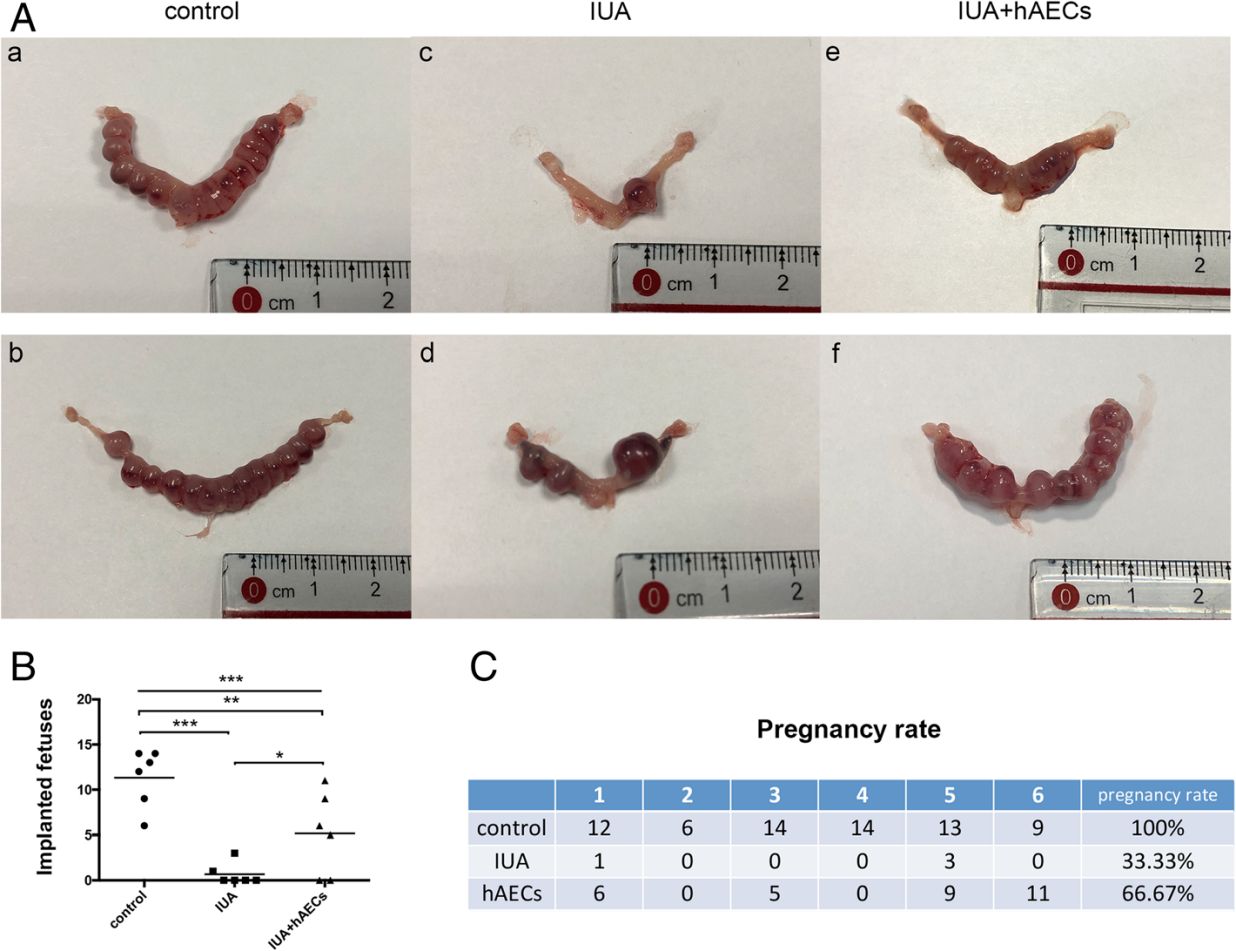
hAECs ameliorated the pregnancy outcomes in the IUA mouse model. **A** (a, b) For normal day-9 pregnant uterine masses, the implanted fetuses were of similar size and shape, lined up in order. (c, d) In IUA uterine horns, the number of fetuses was fewer, and the two uterine horns of one mouse were asymmetric; the sizes of the fetuses varied. (e, f) In the hAEC group, the number of fetuses increased. **B** The fetus size was significantly smaller in the IUA group (0.67 ± 1.211) than in the control group (11.33 ± 3.204), and the litter size of the hAEC-treated group was significantly greater (5.1 ± 4.535) (*n* = 6; *p < 0.05; **p < 0.01; ***p < 0.001; NS, *p* ≥ 0.05). **C** The pregnancy rate of each group was calculated. The pregnancy rate was significantly lower in the IUA group than in the normal control. After treatment with hAECs, the pregnancy rate recovered to 66.67%

Gross examination revealed symmetrical uterine horns and similar fetus sizes in the control group. However, the size of the fetuses varied in the IUA group (Fig. 6A). Moreover, the total number of fetuses of each mouse was significantly higher in the hAEC-treated group (5.17 ± 4.535) than in the IUA group (0.67 ± 1.211) but lower than in the normal control group (11.33 ± 3.204) (Fig. 6B). All mice in the normal control group were pregnant (100%), while the pregnancy rate (66.67%) was higher in the hAEC-treated group than in the IUA group (33.33%) (Fig. 6C).

### hAECs rescue impaired autophagy in endometrial stromal cells in vivo and in vitro

**Fig. 7:**
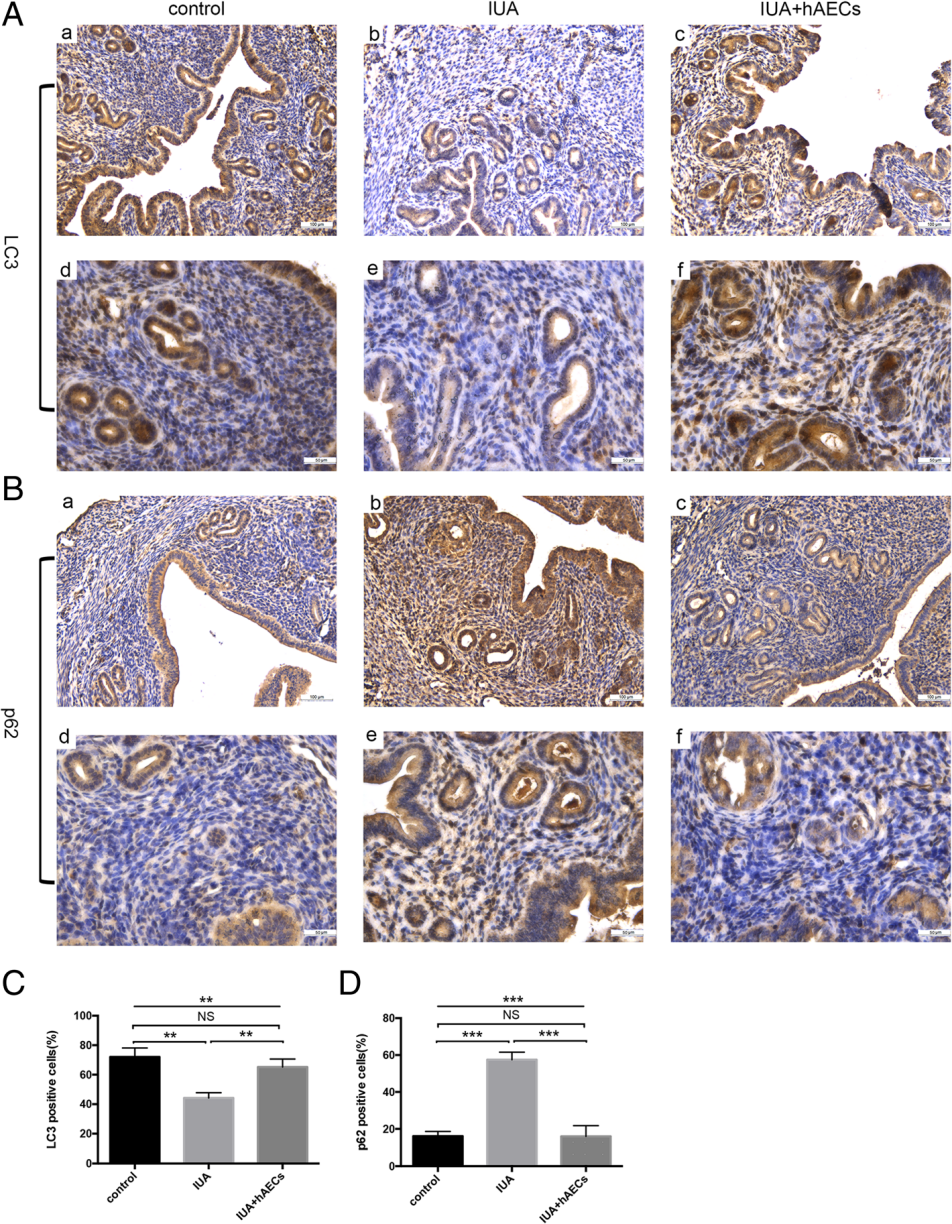
hAECs rescued impaired autophagy in the endometrium. **A** According to IHC staining, the number of LC3-positive cells was reduced in the IUA group and increased in the hAEC-treated group. **B** The number of p62-positive cells was increased in the IUA group and reduced in the hAEC-treated group. **C**, **D** Semi-quantification of LC3 and p62 expression in murine endometrium was calculated as the percentage of positive cells per field (*p < 0.05; **p < 0.01; ***p < 0.001; NS, *p* ≥ 0.05). a–c, scale bar = 100 μm; d–f, scale bar = 50 μm

Autophagy is a programmed cellular degradation process that responds to environmental stress and plays an important role in the periodical alterations of the endometrium [26]. To evaluate the effect of hAEC transplantation on endometrial autophagy, we assessed the expression of LC3, an indicator of autophagy, and p62, an indicator of autophagic flux inhibition. IHC staining demonstrated that the percentage of stromal cells positive for LC3 was significantly higher in the hAEC-treated group than in the IUA group, reaching almost the same rate as that in the control group (Fig. 7A, C). However, the expression of p62 was remarkably higher in the IUA group than in the normal control group and significantly lower in the hAEC-treated group (Fig.7B, D). These results indicate that hAECs were helpful in regulating autophagy in endometrial stromal cells.

Furthermore, we used an in vitro coculture system to confirm whether hAECs regulate autophagy in endometrial stromal cells in vitro. Human endometrial mesenchymal stem cells (hEnSCs) were isolated from menstrual blood and shown to have endometrial stromal cell characteristics [25].

**Fig. 8:**
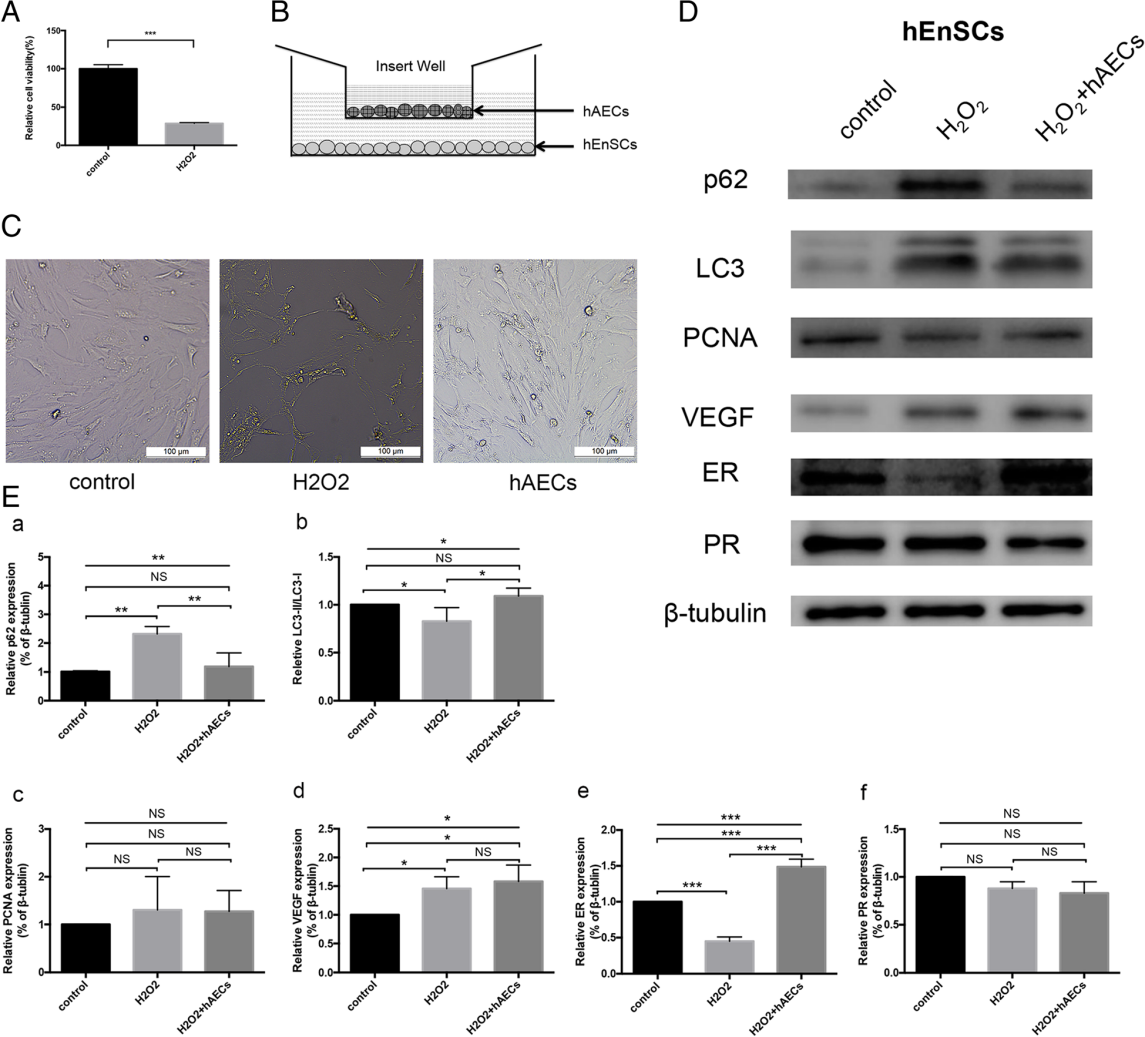
hAECs promoted autophagy in hEnSCs in vitro. **A** The cell viability of H_2_O_2_-treated hEnSCs significantly decreased. **B** hEnSCs were cocultured with hAECs in a Transwell system. **C** After 2.5 h of H_2_O_2_ treatment and another 24 h of culture, hEnSCs shrank severely, but hAEC coculture repaired the cell morphology of hEnSCs damaged by H_2_O_2_. **D** Western blot analysis showed that p62 expression increased significantly in H_2_O_2_-treated hEnSCs and decreased in hEnSCs cocultured with hAECs. The relative expression of LC3-II/LC3-I was decreased in H_2_O_2_-treated hEnSCs and increased in hEnSCs cocultured with hAECs. The expression level of ER was downregulated in H_2_O_2_-treated hEnSCs but upregulated in hEnSCs cocultured with hAECs. The expression of VEGF, PCNA, and PR did not change prominently. **E** The grayscale values of the western blots were evaluated. The ratios of LC3-II/LC3-I were standardized to those of the control group. The protein levels of p62, PCNA, VEGF, ER, and PR were normalized to that of β-tubulin (*n* = 3; *p < 0.05; **p < 0.01; ***p < 0.001; NS, *p* ≥ 0.05)

hEnSCs were incubated with 1 μM H_2_O_2_ for 2.5 h to imitate the impaired endometrium stromal cells [24]. The results showed that the cell viability of hEnSCs decreased after exposure to H_2_O_2_ (Fig. 8A). H_2_O_2_ treatment decreased the number of adherent hEnSCs and increased cell shrinkage; however, after coculture with hAECs, the morphology of H_2_O_2_-treated hEnSCs was restored (Fig. 8B, C).

Next, we investigated the effect of hAECs on the autophagy of hEnSCs by western blotting. Consistent with the IHC results, the protein level of p62 was significantly higher in H_2_O_2_-treated hEnSCs than in the control cells; however, the protein level of p62 in hEnSCs cocultured with hAECs was significantly decreased. In addition, H_2_O_2_ treatment significantly reduced the ratio of LC3-II/LC3-I in hEnSCs, whereas the ratio of LC3-II/LC3-I in hEnSCs cocultured with hAECs dramatically increased (Fig. 8D, E).

Several other proteins related to endometrium repair, including ER, PR, PCNA, and VEGF, were also evaluated by western blotting. The results showed that H_2_O_2_ treatment led to reduced levels of ER expression molecules and that hAECs could restore these expression levels in H_2_O_2_-treated hEnSCs. However, we did not observe similar changes in VEGF, PCNA, and PR expression in these three groups (Fig. 8D, E). These results indicate that hAEC transplantation could at least partly recover the damaged endometrium through autophagy induction.

## Discussion

The aim of this study was to determine whether hAEC transplantation could restore the fertility of an IUA mouse model. Herein, we successfully established an IUA model in female mice by mechanical injury. When IUA occurred, the murine uterus became adherent, the endometrium was thinner, the glands and microvessels decreased, and the fibrotic area increased, resulting in decreased fertility. By transplanting hAECs into IUA mice, the endometrial morphology improved, and the pregnancy rate and litter size increased. Furthermore, we observed autophagy suppression in endometrial stromal cells in IUA mice. Using a coculture system in vitro, we demonstrated that hAECs could activate autophagy in damaged endometrial stromal cells.

We conducted mechanical injury to the murine uterus to imitate uterine curettage. All intrauterine operations were performed by the same experimenter to minimize the bias resulting from the differences in personal techniques. We have previously shown that intraperitoneal hAEC injection improved ovarian function in a mouse model of primary ovarian insufficiency [22]. Different methods, including tail vein injection, intraperitoneal injection, intramuscular injection, and intrauterine injection, have been used for stem cell transplantation to facilitate endometrium repair in animal models [12, 14, 16, 17]. As described in Kilic’s study, hAECs were transplanted into an IUA murine model by intraperitoneal injection immediately after the operation and for three subsequent days [12]. Our cell tracking assay showed that hAECs migrated to the injured uterus, which demonstrated that hAECs homed to the damaged uterus (Fig. 3B). (Linefeed) Autophagy is a cellular degradation process in response to environmental stress that breaks down senescent organelles and provides energy when nutrition is lacking [27]. Autophagy is closely relevant to physiological and pathological activities such as inflammation, apoptosis, cell proliferation, differentiation, and metabolism, and plays an important role in maintaining the endometrial function [28, 29]. Choi et al. collected endometrial samples from premenopausal/nonpregnant women and found that LC3-II gradually increased and reached its peak level at the late secretory phase within the menstrual cycle. These authors also found that autophagy worked in concert with apoptosis to rebuild the endometrium during the menstrual cycle [26]. Tseng et al. analyzed an RNA array of endometrial samples from premenopausal women and found that two autophagy-related genes, γ-aminobutyric acid receptor-associated protein-like 1 (GABARAPL1) and γ-aminobutyric acid receptor-associated protein-like 3 (GABARAPL3), showed the highest expression in the mid-secretory phase and the lowest expression in the late secretory phase [30]. Xu et al. studied the repairing effect of temperature-sensitive heparin-modified poloxamer hydrogel with affinity to keratinocyte growth factor (KGF) in a rat IUA model. These authors demonstrated that LC3-II was decreased and that p62 was increased in the IUA model, but with hydrogel and KGF treatment; LC3-II relatively increased; and p62 decreased. This evidence suggested that endometrial autophagy was suppressed when IUA occurred, and the recovery process included autophagy rescue [24]. Consistent with these findings, we showed that hAECs impaired autophagy in a murine IUA model and that hAECs activated autophagy in endometrial stromal cells in vitro.

Previously, we used a cytokine array assay and reported that concentrated hAEC medium contained abundant cytokines, including autophagy-related factors, such as IL-2, IL-6, and IL-1. IL-2 is an autophagy-activating cytokine [31]. Recombinant IL-2 upregulated autophagy in liver injury in C57BL/6 mice [32]. IL-2 activated autophagy in mouse embryonic fibroblasts and primary lung fibroblasts by regulating ATG5 and *Beclin1* [33]. Our Transwell coculture assay suggested that the autophagy-inducing effect of hAECs could occur through paracrine effects. However, whether hAECs induce autophagy by secreting cytokines, such as IL-2, should be further elucidated.

In the clinic, patients with Asherman syndrome are more likely to be infertile and suffer more pregnancy complications after surgical treatment. For example, the thin endometrium of IUA uteruses reduces the chance of pregnancy, and the low oxygen content impairs endometrial receptivity [34]. Thus, adhesiolysis may not fully rescue the fertility of Asherman syndrome patients. For further fertility improvement, it is necessary to create a better endometrial environment in multiple aspects, including blood supply and oxidative stress. Therefore, we analyzed several molecules relevant to endometrial recovery in a murine model.

PCNA is responsible for accurate DNA duplication [35]. Niklaus et al. compared endometrial samples from reproductive-age women and found that in both stroma and epithelial tissues, PCNA was most abundant at the proliferative phase but decreased at the secretory phase [36]. In our study, hAECs significantly increased PCNA expression in the murine endometrium, indicating that hAECs might improve proliferation in the endometrium.

VEGF is expressed mostly during the menstrual period and proliferative phase and is relevant to the maintenance and formulation of microvessels and the reconstruction of endometrial tissue [37, 38]. Chen et al. found that hormone replacement therapy (HRT) combined with hysteroscopic adhesiolysis significantly increased endometrial VEGF expression and MVD in IUA patients. Additionally, those who had a better curative effect had higher VEGF expression and denser microvessels than those who responded poorly to the treatment. In our study, hAECs increased VEGF expression and MVD in the IUA model, indicating the angiogenesis effect of hAECs, which might facilitate endometrial injury recovery.

ER is a nuclear transcription factor that promotes metabolism and proliferation in endometrial cells in combination with estrogen [39]. ER expression is significantly increased in the repaired endometrium of allogeneic UCMSC therapy-treated patients [40]. Consistent with this finding, hAECs increased ER expression in damaged murine endometrium. Thus, the status of ER might regulate endometrial injury repair.

Recent studies have shown that stem cells could serve as a therapeutic agent for IUA caused by endometrial injury [41]. In comparison with other stem cells, hAECs derived from human amnion, a type of medical waste, have a sufficient source and can be amplified easily in vitro. Some ethical problems related to embryonic stem cells could be avoided. Additionally, hAECs have low immunogenicity and lack tumorigenesis properties. Thus, hAECs have these advantages for broad application in regeneration medicine.

Here, we report for the first time that hAECs have the potential to restore fertility in an IUA mouse model and that hAECs promote endometrial injury repair by activating the autophagy pathway. Further studies should explore whether the autophagy pathway participates in angiogenesis and whether hAEC treatment combined with an autophagy inhibitor would improve endometrial regeneration.

## Conclusion

The present study demonstrates that intraperitoneal transplantation of hAECs improved endometrium morphology in an IUA mouse model, contributing to a thicker endometrium, more glands, and less fibrotic area in an injured endometrium. hAECs also facilitated endometrial stromal cell proliferation and angiogenesis in an IUA model and promoted pregnancy in an IUA mouse model. Finally, hAECs promoted endometrial injury repair by activating the autophagy pathway.

## Data Availability

Not applicable.

## Abbreviations

ATG: Autophagy-related gene
E_2_: 17β-Estradiol
ESCs: Endometrial stromal cells
ER: Estrogen receptor
H&E: Hematoxylin and eosin
hAECs: Human amniotic epithelial cells
hEnSCs: Human endometrial mesenchymal stem cells
IUA: Intrauterine adhesion
IUD: Intrauterine device
MAPLC3/LC3: Microtubule-associated protein light chain 3
MVD: Microvascular density
PCNA: Proliferating cell nuclear antigen
PR: Progesterone receptor
VEGF: Vascular endothelial growth factor
vWF: von Willebrand factor
